# Larger Workplaces, People-Oriented Culture, and Specific Industry Sectors Are Associated with Co-Occurring Health Protection and Wellness Activities

**DOI:** 10.3390/ijerph15122739

**Published:** 2018-12-04

**Authors:** Aviroop Biswas, Colette N. Severin, Peter M. Smith, Ivan A. Steenstra, Lynda S. Robson, Benjamin C. Amick

**Affiliations:** 1 Institute for Work & Health, Toronto, ON M5G 2E9, Canada; cseverin@iwh.on.ca (C.N.S.); psmith@iwh.on.ca (P.M.S.); isteenstra@morneaushepell.com (I.A.S.); lrobson@iwh.on.ca (L.S.R.); bamickii@fiu.edu (B.C.A.); 2 Dalla Lana School of Public Health, University of Toronto, Toronto, ON M5T 3M7, Canada; 3 Centre of Occupational and Environmental Health, Monash University, Melbourne, VIC 3004, Australia; 4 Morneau Shepell, Toronto, ON M5S 3A9, Canada; 5 Florida International University, Miami, FL 33199, USA

**Keywords:** workforce demographics, health promotion, injury prevention, occupational health

## Abstract

Employers are increasingly interested in offering workplace wellness programs in addition to occupational health and safety (OHS) activities to promote worker health, wellbeing, and productivity. Yet, there is a dearth of research on workplace factors that enable the implementation of OHS and wellness to inform the future integration of these activities in Canadian workplaces. This study explored workplace demographic factors associated with the co-implementation of OHS and wellness activities in a heterogenous sample of Canadian workplaces. Using a cross-sectional survey of 1285 workplaces from 2011 to 2014, latent profiles of co-occurrent OHS and wellness activities were identified, and multinomial logistic regression was used to assess associations between workplace demographic factors and the profiles. Most workplaces (84%) demonstrated little co-occurrence of OHS and wellness activities. Highest co-occurrence was associated with large workplaces (odds ratio (OR) = 3.22, 95% confidence interval (CI) = 1.15–5.89), in the electrical and utilities sector (OR = 5.57, 95% CI = 2.24–8.35), and a high people-oriented culture (OR = 4.70, 95% CI = 1.59–5.26). Promoting integrated OHS and wellness approaches in medium to large workplaces, in select industries, and emphasizing a people-oriented culture were found to be important factors for implementing OHS and wellness in Canadian organizations. Informed by these findings, future studies should understand the mechanisms to facilitate the integration of OHS and wellness in workplaces.

## 1. Introduction

The workplace is a social determinant of health, with employment and working conditions linked to a range of health, functioning, and quality-of-life outcomes [1,2]. Work-related injuries and illnesses are associated with morbidity and substantial financial and social costs, and health hazards from work can also impact people’s personal lives and lifestyle [3,4]. Studies also show that lifestyle risk factors (e.g., being a smoker, stressful lives outside of work, being obese, and heavy alcohol use) can increase the likelihood of sustaining workplace injuries more so than among those without such risk factors [5,6]. North American employers are required to provide occupational health and safety (OHS) activities that minimize negative health effect due to worker exposures to job-related risks and hazards. In comparison, workplace health promotion or wellness activities are voluntarily provided by some employers to improve worker wellbeing through health behavior changes and are shown to have short and long-term health and productivity benefits [7,8].

There has been a shift in thinking about how workplaces can better integrate safety into the overall wellbeing of their workforce [9]. Wellness and OHS programs share the goal of protecting and improving worker health and given these overlaps it makes sense to integrate both. Integrating OHS and wellness activities is expected to have greater effects on health, safety, and wellbeing than if the activities operated separately from each other [10,11]. This approach, commonly referred to as Total Worker Health^®^ in the US, is widely endorsed by international health and labor agencies [12,13,14] with the consensus that it will lead to improvements in the long-term well-being of workers and their families, and reduce pressures on healthcare and social security systems [13,14].

Several studies have demonstrated links between the characteristics of workplaces (workplace demographic factors) and the implementation and integration of OHS and wellness activities. For example, the manufacturing sector reports a higher number of OHS and wellness activities than other sectors [15,16], and smaller workplaces are likely to offer fewer OHS and wellness programs than larger organizations [15,17,18]. Examining workplaces in the US Midwest, McLellan et al. found leadership support and having an OHS committee to be important contributors to implementing integrated approaches [19]. Tremblay et al. examined Massachusetts employers and found a high degree of coordinated activities among unionized workplaces and in construction, healthcare, manufacturing, and entertainment industries [18]. These and other studies are limited in their focus on small workplaces [17,19] and sampled few larger workplaces [18]. A greater focus on medium to large in addition to smaller workplaces can further uncover factors enabling integrated activities as larger workplaces are likely to have more resources to support these activities [20]. Furthermore, much of the research examining relationships between workplace factors and the implementation of OHS and wellness have focused on US workplaces and little is known about the extent that these activities co-occur in Canadian workplaces. To inform research and policy recommendations towards the widespread adoption of integrated worker health approaches in Canada, research evidence is needed to understand the extent that OHS and wellness activities co-occur as a necessary first step towards identifying the current status quo and the workplace factors that can be amenable targets for integrated approaches in the future.

The objective of this study was to explore the workplace demographic factors associated with the concurrent implementation of OHS and wellness activities in Canadian workplaces. The study has two research questions (RQ): RQ1—“What is the extent that OHS and wellness activities co-occur in workplaces?” RQ2—“Are there associations between workplace demographic characteristics and the co-occurrence of OHS and wellness activities?” These questions were explored in a cross-section of a large, heterogenous sample of small, medium, and large workplaces in Ontario, Canada. Informed by evidence from US studies, we hypothesized that large and unionized workplaces in specific industry sectors have a higher co-occurrence of OHS and wellness activities than workplaces with other demographic characteristics. 

## 2. Materials and Methods

### 2.1. Data Sources

This study analyzed data from the Ontario Leading Indicators Project (OLIP), a cross-sectional survey conducted by researchers at the Institute for Work & Health from 2011 to 2014 in partnership with health and safety associations in Ontario, Canada. The aim of the OLIP study was to identify leading indicator measures for workplaces to improve their health and safety performance before injuries and illnesses occur. Study details are available elsewhere [21]. Briefly, OHS and wellness data were collected in collaboration with four OHS associations representing employers from most labor sectors in Ontario. The target population consisted of organizations registered with the Workplace Safety & Insurance Board (WSIB), an organization responsible for workers’ compensation to approximately 62.5% of Ontario’s workforce [22]. Workplace Safety & Insurance Board compensation coverage is optional for certain workers such as independent contractors, sole proprietors, and partners in partnerships. Organizations included in the study analysis had at least one full-time employee in the following industries: education, electrical and utilities, agriculture, manufacturing, municipal, healthcare, service, pulp and paper, forestry, and mining. Other industries were not examined.

### 2.2. Sampling and Recruitment

Workplaces were identified by random stratified sampling based on the following variables: industrial sector, geographic region, and size. Study recruitment took place from 15 March 2011, to 27 August 2012, which began with the OLIP study’s health and safety association partners making initial contact with organizations to solicit their interest. If an organization consented to take part in the study, the person most knowledgeable about health and safety at their organization completed an online English-language questionnaire. Respondents were also given the option to complete the questionnaire by mail or phone. Questionnaires were administered to each respondent in a random sequence. Three to ten follow-up e-mails or phone calls were sent to remind participants to complete questionnaires. The study was approved by the University of Toronto’s research ethics board (protocol 25363).

### 2.3. Measures

#### 2.3.1. Independent Variables: Workplace Demographic Characteristics

The independent variables used in the analysis were workplace demographic characteristics chosen a priori from the available literature as they were found to be linked to the co-occurrence of OHS and wellness activities. The following variables were examined, workplace size (four categories; small and without a Joint Health and Safety Committee, JHSC (reference category)), union status (unionized, non-unionized (reference category), don’t know), industry sector (eight sectors; manufacturing sector (reference category)). Workplace culture factors previously found to be associated with OHS and wellness implementation [19,23] were examined using a measure of workplace health and safety leadership (lowest = 1 (reference category), highest = 4), and a measure of people-oriented culture (lowest = 1 (reference category), highest = 4) (Supplementary Table S1).

Union status (i.e., at least part of the workforce is represented by a union) was self-reported in the OLIP questionnaire. Questionnaire responses were linked to corresponding 2009 WSIB administrative records to obtain additional information on a workplace’s industry sector classification. Workplace size was self-reported and categorized according to Statistics Canada classifications [24] as follows, small workplaces had 1 to 99 employees, medium workplaces had 100 to 499 employees, and large workplaces had ≥500 employees. Workplace size was a proxy of OHS infrastructure and accordingly, small workplaces were further classified by whether they reported having a JHSC (consists of labor and management representatives regularly meet to deal with health and safety issues). This categorization was based on the mandatory Ontario labor requirement that workplaces with >20 employees must have a JHSC. An organization’s leadership support and culture were examined using two subscales selected a priori from the Organizational Policies and Practices questionnaire [25,26]: Health and Safety Leadership (six items) and People-Oriented Culture (four items). Participants rated the extent their organization achieved these subscales on a five-point scale from 0% (never) to 100% (always). Each subscale item was averaged to a score ranging from 0 (low) to 4 (high).

#### 2.3.2. Occupational Health and Safety and Wellness Activities

A workplace’s OHS activities were measured by the availability of factors related to safe and effective OHS performance as an alternative measure to the number of OHS activities offered by workplaces as these can vary by working conditions and industry sector. For example, workplaces in high hazard industries are likely to overrepresent higher OHS activities than other workplaces. Occupational health and safety performance was measured using the Institute for Work & Health’s Organizational Performance Metric (IWH-OPM) tool, which has been shown to be predictive (i.e., valid and reliable) of future injury and illness rates [27,28]. The eight-item IWH-OPM tool was developed by a consensus process among a team of researchers and health and safety professionals [28]. For each IWH-OPM item, respondents rated the percent of time that certain practices occurred in their workplace, from 1 (0–20%) to 5 (80–100%). Scores for each item were summed to estimate a total OHS performance score, with a highest score of 40 (a score of 5 for all items) indicating that all eight OHS practices took place most to all the time in a workplace.

A workplace’s wellness activities were assessed by dichotomous (“yes” or “no”) responses to the question: “during the last 12 months, did your company offer any of the following programs to employees and/or their families?” The range of wellness activities were selected from the literature and the Centers for Disease Control and Prevention’s Workplace Health Model [29,30]. Wellness programs included screenings (blood pressure, diabetes, cholesterol, and cancer), smoking cessation classes, physical activity and/or fitness classes, and educational resources. Wellness policies included flexible work hours to engage in wellness activities, encouraging fitness breaks, and healthy food choices. Supportive environments for wellness activities included providing shower facilities, signage to encourage stair use, and on-site fitness facilities or walking trails. The total number of wellness activities was derived from a total score of 25 possible options.

#### 2.3.3. Outcome Variable: Co-Occurrence of Occupational Health and Safety and Wellness Activities

Occupational health and safety performance and wellness activities were examined as continuous variables and categorized into profiles based on similar response probability patterns for the total scores of each activity.

### 2.4. Analyses

Statistical analyses were conducted using commercially-available statistical software, SAS v. 9.4 (SAS Institute Inc., Cary, NC, USA) [31] and Mplus v. 8.0 (Muthén & Muthén, Los Angeles, CA, USA) [32], and tests were two-sided with significance set at *p* = 0.05. Workplaces represented by OLIP questionnaire responses were statistically weighted to permit inferences from the sample to a comparable population of Ontario organizations based on strata of workplace size, region, and industry sector. For RQ1, mean values of OHS and wellness activities were compared separately according to workplace size, union status, industry sector, health and safety leadership, and people-oriented culture. Analysis of variance was used to examine differences in the mean OHS and wellness activities scores. Workplaces were assigned to ‘profiles’ based on the probability that they had similar numbers of OHS and wellness activities to other workplaces using Mplus’s latent profile analysis function. The latent profile analysis statistical technique aims to recover hidden groups from observed data, similar to clustering techniques, but is more flexible because the approach is based on an explicit model of the data, and accounts for the fact that recovered groups are uncertain [33]. Data on OHS performance scores and number of wellness activities were transferred from SAS to Mplus and analyzed as continuous variables in a mixture model with sample weights. Several models were fit with increasing numbers of profiles (one profile, two profiles, three profiles etc.). A decision on the most suitable number of profiles fitting the data was made by inspecting model-fit statistics for the Lo–Mendell–Rubin adjusted likelihood ratio test. The Lo–Mendell–Rubin test had a *p*-value of 0.58 when comparing four profiles to three profiles, suggesting that three profiles sufficiently modelled the data. For RQ2, associations between the latent profile groups and workplace demographic characteristics (independent variables) were estimated using multinomial logistic regression by transferring latent profile probability data generated from Mplus back into SAS and matching them to corresponding data from individual survey respondents. The odds of a co-occurrence profile associated with a workplace characteristic of interest compared to the odds of the lowest co-occurrence profile and a reference workplace characteristic (e.g., a small workplace without a JHSC) were described as odds ratios (OR) and 95% confidence intervals (CIs).

## 3. Results

### 3.1. Descriptive Statistics

Representatives from 1692 organizations responded to the OLIP survey from 7285 approached (23.2% response rate). Excluded from the analysis were respondents with missing data for the variables of interest or if respondents did not indicate “none of the above” if indicating the absence of wellness activities rather than overlooking the question, to leave a final analytical sample of 1285 responses. Most respondents were managers (36%) and had >5 years of experience at their organization (70%). Table 1 describes the workplace demographic characteristics. Most workplaces were classified as small and without a JHSC (53%), non-unionized (90%), and in the manufacturing (30%) or service (54%) sectors. The most frequently reported wellness programs were employee assistance programs (EAPs) (15%), physical activity and/or fitness programs (14%), and stress reduction programs (13%), while different health screening and education programs were reported the least. The most frequent wellness policy was flexible hours (40%) followed by working from home (14%). Onsite shower facilities (15%) was the most frequently reported environmental support.

### 3.2. Co-Occurrence of Occupational Health and Safety and Wellness Activities

**Figure 1:**
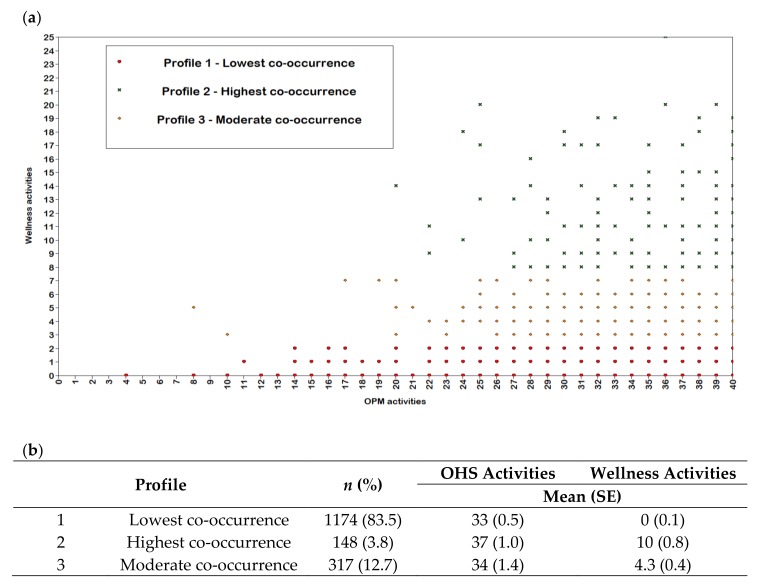
Co-occurrence of workplace occupational health and safety (OHS) and wellness activities based on workplaces with similar profiles. (**a**) Co-occurrence profiles (lowest co-occurrence (red circle), moderate co-occurrence (orange diamond), highest co-occurrence (green cross)) and (**b**) descriptive characteristics of the profiles. SE, Standard Error.

The number of OHS and wellness activities were found to be poorly correlated (Pearson’s *r* = 0.14) indicating that the co-occurrence of both was low among the surveyed workplaces. Figure 1 shows common profiles of co-occurrent OHS and wellness activities according to latent profile analysis. Three distinct profiles of workplace OHS and wellness activities were identified. Profile 1 indicated the group of workplaces with the lowest occurrence of OHS and wellness activities (84% of responses), with a mean of 33 OHS activities and no wellness activities. Profile 2 indicated the group of workplaces with the highest occurrence of OHS and wellness activities (4% of responses), with a mean of 37 OHS activities and 10 wellness activities. Profile 3 indicated the group of workplaces with a moderate co-occurrence of OHS and wellness activities (13% of responses), with a mean of 34 OHS activities and four wellness activities.

### 3.3. Asociations between Workplace Demographic Characteristics and the Co-Occurrence of Occupational Health and Safety and Wellness Activities

Table 2 shows associations between workplace demographic characteristics and the co-occurrence profiles of OHS and wellness activities. Increasing workplace size was associated with greater odds of a workplace being classified in the highest (profile 2) and moderate (profile 3) co-occurrence profiles compared to small workplaces without a JHSC. Large workplaces were estimated to have the greatest odds of being in the highest co-occurrence profile compared to small, non-JHSC workplaces (OR = 3.22, 95% CI = 1.15–5.89). Compared to small, non-JHSC workplaces, medium workplaces were most likely to be classified in the moderate co-occurrence profile (profile 3) (OR = 4.71, 95% CI = 1.42–8.74), followed by large workplaces (OR = 2.22, 95% CI = 1.05–4.52). Unionized workplaces were more likely to be a member of the highest co-occurrence profile (profile 2) (OR = 1.52, 95% CI = 0.48–4.88) or moderate co-occurrence profile (OR = 1.03, 95% CI = 0.33–3.27) compared to non-unionized workplaces, although these estimates were not statistically significant. Workplaces in the electrical and utilities (OR = 5.57, 95% CI = 2.24–8.35) and municipal (OR = 5.52, 95% CI = 0.91–8.43) industry sectors were most likely to be classified as a highest occurrence profile compared to the manufacturing sector, although the association with the municipal sector was not statistically significant. Workplaces in the electrical and utilities (OR = 7.67, 95% CI = 2.46–10.50) and municipal sectors (OR = 6.97, 95% CI = 1.80–9.06) were also most likely to be classified in the moderate co-occurrence profile. No statistically significant associations were found between scores for health and safety leadership and the likelihood of highest and moderate co-occurrence profiles. Workplaces rated highest for people-oriented culture were likely to be classified in the highest co-occurrence profile (OR = 4.70, 95% CI = 1.59–5.26) compared to lowest-rated workplaces. No statistically significant associations were found between people-oriented culture ratings and the moderate co-occurrence profile.

## 4. Discussion

This study surveyed a large and diverse sample of workplaces to examine the extent that OHS and wellness activities co-occur and identified the workplace characteristics most likely to be associated with the co-occurrence of the activities. Most workplaces surveyed reported having few OHS and wellness activities. Large workplaces, those in the electrical and utilities sector, and with a high rating for people-oriented culture were factors most associated with a high co-occurrence of OHS and wellness activities. Large and medium-size workplaces, those in the electrical and utilities, and municipal sectors were associated with a moderate co-occurrence of activities.

This study found a fewer number of wellness activities in Ontario workplaces compared to US studies where at least three-quarters of workplaces reported one or more wellness activity [17,18]. This difference may be due to the fewer incentives for Canadian employers to invest in wellness activities as most medically-necessary services are covered by Canada’s public healthcare system. Furthermore, employer contributions to healthcare costs are comparatively modest. The higher number of EAPs compared to other wellness activities is unsurprising as small workplaces represented the highest proportion of respondents in the study. The outsourcing of resources to EAPs can represent a better investment for small workplaces compared to more costly investments in onsite wellness activities [20]. Small organizations also experience several obstacles to implementing wellness activities such as constraints in assigning resources and dedicated staff for wellness initiatives, perceiving a lack of employee interest in participating in wellness activities, or have poor access to health promotion resources and wellness providers [20]. While stress management, physical activity promotion, and flexible hours were reported frequently, others such as healthy food choices and shower facilities were less frequently reported. This suggests that workplaces might be primarily focusing on encouraging their workers to change their own behaviors. Yet only focusing on changing individual behaviors is unlikely to lead to meaningful worker health improvements since a small percentage of workers participate in wellness activities without workplace policies and environmental supports also in place [3]. 

A small proportion of workplaces indicated a moderate or high co-occurrence of OHS and wellness activities, and similar findings have also been reported elsewhere [17,18]. Medium and large organizations were more likely to provide moderate to high co-occurrence of OHS and wellness activities compared to small workplaces, while having a JHSC did not show meaningful differences in wellness activities among small workplaces. This suggests that medium- to large-size workplaces are more likely than small workplaces to have the resources and supports in place to promote these efforts concurrently. Workplaces in the service, agriculture, and pulp and paper sectors were least likely to report co-occurring activities, while the electrical and utilities and municipal sectors were most likely. These differ from findings previously reported among employers in Massachusetts [18]. Whether the differences in our study reflect true differences in the employer population in Ontario compared to Massachusetts or reflects selection bias between the two studies, requires closer inspection. Further research is also needed to examine the workplace practices in sectors pertaining to low and high OHS and wellness activities to better understand the reasons for the implementation differences in Canada. Our findings also suggest that a people-oriented culture can at the very least support a higher implementation of wellness activities. Employer efforts to create a workplace culture of trust and respect might enhance workers’ receptivity and openness to messages and programs designed to change behaviors and improve health [34,35].

Some limitations need to also be acknowledged when interpreting these findings. First, this was a cross-sectional study and causal relationships cannot be directly inferred. Second, the response rate was low, although the study’s large initial sample size can facilitate the detection of more robust and reproducible statistical relationships than previous research with smaller sample sizes. We also reduced nonresponse bias by statistically weighting all modelling estimates to infer responses from a population of comparable organizations in the Ontario labor market. Third, our findings are only generalizable to the industry sectors we sampled and future studies need to examine how our findings relate to other industries such as the finance, information, professional, and entertainment sectors. Fourth, our use of a self-reported survey is prone to recall and social desirability biases. Differential measurement error is also possible across OHS and wellness activities. Respondents to the survey were selected based on their knowledge of OHS activities in the workplace, not on wellness activities. As such, it is possible that respondents could estimate OHS activities more accurately than wellness activities. Fifth, it is possible that some wellness activities were counted more than once if they were also provided as part of an EAP service (e.g., education, risk management tools, and self-care materials). Nonetheless, we conducted a sensitivity analysis and found that co-occurrence profiles did not meaningfully change when wellness activities that might be part of an EAP were removed. Lastly, the OLIP survey was not designed to collect detailed wellness information or the extent that these are integrated and coordinated with OHS activities.

Integrated OHS and wellness activities are widely promoted as an effective approach to chronic disease prevention [36]. This is partly explained by the emergence of evidence supporting the idea that workplace factors contribute to adverse health outcomes traditionally considered to be unrelated to work (such as stress, heart disease, and mental health) [10,11,37]. While distinguishing workplaces by their implementation of OHS and wellness activities may not reflect a truly integrated worker health approach [23], our findings provide a better understanding of the workplace factors associated with having suitable resources to support an integrated approach in the Canadian labor market. In 2016, a panel report from the National Institute of Health Pathways to Prevention Workshop identified small workplaces as a priority area for supporting integrated approaches through Total Worker Health^®^ [38]. However, as our study and others have shown [15,16,17,18], there is a lack of demonstrated effectiveness in smaller workplaces in the concurrent adoption of health protection and wellness programming. Smaller workplaces might not integrate their OHS and wellness resources not because of a lack of support or motivation per se, but because of a lack of resources, including personnel, which might make it challenging to just perform traditional OHS hazard control alone [39]. Findings show that larger workplaces, with a people-oriented culture, and in the electrical and utilities, and municipal sectors are associated with more health protection and wellness resources that can be streamlined into integrated approaches. The next logical step is to examine intermediate and long-term health and productivity changes for these workplaces expected to benefit the most from co-occurring and integrated OHS and wellness activities. Subsequent findings can inform studies that are extended or scaled to other industries and smaller workplaces. Actionable recommendations whereby Canadian workplaces can integrate their existing OHS and wellness activities and ingrain these within a workplace’s culture is also an area of research that needs to be explored further. Integration can also be enabled by an integrated safety and wellness committee, shared budgets and resources, and incentivizing employees in health protection and health promotion efforts [23].

## 5. Conclusions

This study provides valuable information on the co-occurrence of OHS and wellness activities and identifies workplace demographic factors most associated with their implementation in Canadian workplaces. Large workplaces, those in the electrical and utilities sector, and with a high rating for people-oriented culture are factors strongly related to the implementation of both OHS and wellness and might benefit most from integrated worker health activities. Future research needs to understand how to facilitate the uptake of OHS and wellness activities in workplaces with fewer concomitant organizational resources to increase OHS and wellness implementation. Furthermore, our findings need to be verified in other workplace contexts that were not explored in this study, and the factors that influence organizational change and worker participation need also to be better understood. Finally, it will be important to understand how to streamline OHS and wellness activities in workplaces for an integrated worker health approach.

## Figures and Tables

**Table 1:** Characteristics of surveyed workplaces (*n* = 1285). Statistically-weighted values described.

Characteristic	*n*	% or M	SD
Workplace size			
Small (<100 employees) without a JHSC	171	53.2	4.1
Small (<100 employees) with a JHSC	511	28.3	3.3
Medium (100 to 499 employees)	267	8.0	1.8
Large (>500 employees)	81	1.9	0.8
Union status			
Non-unionized	964	90.1	1.9
Unionized	304	5.9	0.9
Don’t know	10	4.1	4.2
Industry sector			
Manufacturing	440	30.4	1.4
Service	412	53.6	2.9
Healthcare	197	4.4	0.6
Agriculture	161	10.2	1.4
Education	81	0.8	0.1
Municipal	62	0.4	0.1
Pulp and paper	24	0.1	<0.1
Electrical and utilities	13	0.1	<0.1
Occupational health & safety performance (IWH-OPM, range: 1 to 5)			
Formal safety audits at regular intervals		3.3	15.3
Organization values ongoing safety improvement		4.3	9.4
Safety as important as work production and quality		4.4	9.5
Workers and supervisors have information to work safely		4.5	8.5
Employees always involved in health and safety decisions		4.3	9.3
Those in charge of safety have authority to make necessary changes		4.5	9.1
Positive recognition for those who act safely		4.0	11.9
Everyone has the tools and/or equipment to complete work safely		4.6	7.9
Workplace wellness activities			
Flexible work hours for wellness	484	39.8	2.2
Have onsite shower facilities	365	15.0	2.2
Employee assistance programs	394	14.6	2.5
Physical activity and/or fitness programs	272	14.2	2.4
Programs to prevent/reduce stress	226	12.6	2.4
Self-care books/tools	210	11.9	2.1
Nutrition education	221	11.7	2.4
Education on balancing work and family	164	11.4	2.2
Provide or encourage fitness breaks	158	8.1	1.7
Have fitness or walking trails on site	133	6.4	1.5
Health risk assessment	87	5.8	1.9
Smoking cessation classes/counselling	184	5.7	1.5
Weight management classes/counselling	115	5.2	1.9
Screenings for high blood pressure	83	5.2	1.6
Alcohol or drug abuse support programs	173	4.3	0.9
Cholesterol reduction education	68	4.1	1.4
Screenings for cholesterol level	35	3.2	1.6
Screening for diabetes	30	2.4	1.3
Chronic disease management programs	66	2.3	0.9
Promotions/discounts to encourage health food choices	158	2.2	0.9
Label health food choices in cafeteria	76	2.2	1.0
Nurse advice line	41	1.9	0.8
Screenings for any form of cancer	24	1.7	0.9
Have signage to encourage people to use the stairs	41	1.7	0.9
HIV/AIDS education	22	0.4	0.1

M, Mean; SD, Standard Deviation; JHSC, Joint Health and Safety Committee; IWH-OPM, Institute for Work & Health-Organizational Performance Metric tool; HIV/AIDS, Human Immunodeficiency Virus/Acquired Immune Deficiency Syndrome.

**Table 2:** Associations between workplace demographic characteristics and the co-occurrence of occupational health and safety and wellness activities (*n* = 1285) ^1^.

Characteristic	Profile 2	Profile 3
	Highest Co-Occurrence	Moderate Co-Occurrence
	OR (95% CI)	
Workplace size		
Small (<100 employees) without a JHSC	Reference	Reference
Small (<100 employees) with a JHSC	0.32 (0.05–2.19)	1.48 (1.15–4.25)
Medium (100 to 499 employees)	2.76 (0.43–3.59)	4.71 (1.42–8.74)
Large (>500 employees)	3.22 (1.15–5.89)	2.22 (1.05–4.52)
Union status		
Non-unionized	Reference	Reference
Unionized	1.52 (0.48–4.88)	1.03 (0.33–3.27)
Industry sector		
Manufacturing	Reference	Reference
Agriculture	1.00 (0.11–9.20)	0.78 (0.46–1.50)
Pulp and paper	0.50 (0.11–2.21)	0.51 (0.10–2.70)
Education	0.74 (0.15–3.67)	4.90 (0.28–8.77)
Electrical and utilities	5.57 (2.24–8.35)	7.97 (2.46–10.50)
Municipal	5.52 (0.91–8.43)	6.97 (1.80–9.06)
Healthcare	1.76 (0.68–4.56)	2.12 (0.72–6.28)
Service	0.13 (0.03–0.59)	1.87 (0.73–4.80)
Health and safety leadership		
1 (low)	Reference	Reference
2	1.77 (0.25–2.66)	0.52 (0.12–2.24)
3	5.19 (0.95–7.52)	0.50 (0.15–1.69)
4 (high)	4.77 (0.73–5.99)	0.60 (0.21–1.74)
People-oriented culture		
1 (low)	Reference	Reference
2	1.63 (0.96–2.40)	3.59 (0.77–6.88)
3	1.73 (2.20–4.41)	4.63 (0.93–6.02)
4 (high)	4.70 (1.59–5.26)	2.77 (0.62–5.42)

^1^ Reference profile category: Profile 1 (lowest co-occurrence). Reference, OR = 1.00; OR, odds ratio; CI, confidence interval; JHSC, Joint Health and Safety Committee.

## Supplementary Materials

The following are available online at http://www.mdpi.com/1660-4601/15/12/2739/s1, Table S1: Ontario Leading Indicators Project (OLIP): survey variable names.
