# Supplementary material for: Larger Workplaces, People-Oriented Culture, and Specific Industry Sectors Are Associated with Co-Occurring Health Protection and Wellness Activities
Source: Int J Environ Res Public Health. 2018 Dec 4;15(12):2739. doi: 10.3390/ijerph15122739 (PMC6313504; doi:10.3390/ijerph15122739)
Supplement: Supplementary file 1 [file ijerph-15-02739-s001.pdf]

Supplementary Table S1. Ontario Leading Indicators Project (OLIP): survey variable names

The following is selection of variables from the larger OLIP questionnaire used for study analysis.

<u>Organizational Performance (Institute for Work & Health's Organizational Performance Measure, IWH-OPM)</u>

We would like to ask you to evaluate your company's Occupational Health & Safety practices. Please rate the percent of time that each practice takes place.

		**0-20%**	**20-40%**	**40-60%**	**60-80%**	**80-100%**
Item 1	Formal safety audits at regular intervals are a normal part of our business.	1 O	2 O	3 O	4 O	5 O
Item 2	Everyone at this organization values ongoing safety improvement in this organization.	O	O	O	O	O
Item 3	This organization considers safety at least as important as production and quality in the way work is done.	O	O	O	O	O
Item 4	Workers and supervisors have the information they need to work safely.	O	O	O	O	O
Item 5	Employees are always involved in decisions affecting their health and safety.	O	O	O	O	O
Item 6	Those in charge of safety have the authority to make the changes they have identified as necessary.	O	O	O	O	O
Item 7	Those who act safely receive positive recognition.	O	O	O	O	O
Item 8	Everyone has the tools and/or equipment they need to complete their work safely.	O	O	O	O	O

Health and Safety Leadership

Think about the role top management plays in supporting health and safety practices at your company. Please rate the extent to which your company achieves these practices from “Never” or 0% of the time, to “Always”, or 100% of the time. If not applicable, please select “Never”.

		**Never (0%)**	**Sometimes (25%)**	**Half of the time (50%)**	**Most of the time (75%)**	**Always (100%)**
Item 1	Top management is actively involved in the safety program.	1 O	2 O	3 O	4 O	5 O
Item 2	The safety manager (or, the person in charge of health & safety) receives support from top management.	O	O	O	O	O
Item 3	Your company spends time and money on improving safety performance.	O	O	O	O	O
Item 4	Your company considers safety to be equally important as production and quality in the way work is done.	O	O	O	O	O
Item 5	Your company analyzes injury and illness data (e.g., claims data, first aid logs) to identify causes and target solutions.	O	O	O	O	O
Item 6	The safety program or committee has the responsibility, authority and resources to identify and address safety problems.	O	O	O	O	O

People-Oriented Culture

Think about your company's work environment. Please rate the extent to which your company achieves these practices from "Never", or 0% or the time, to "Always", or 100% of the time. If not applicable, please select "Never".

		Never (0%)	Sometimes (25%)	Half of the time (50%)	Most of the time (75%)	Always (100%)
Item 1	Employees are involved in decisions affecting their daily work.	1 O	2 O	3 O	4 O	5 O
Item 2	Working relationships are cooperative.	O	O	O	O	O
Item 3	There is a high level of trust in the employee/employee relationship at your company.	O	O	O	O	O
Item 4	Communication is open, and employees feel free to voice concerns and make suggestions.	O	O	O	O	O

Wellness activities

These questions ask about health promotion programs. Please provide your best answer to these questions.

During the last 12 months, did your company offer any of the following programs to employees and/or their family? *(please select all that apply)*

Item 1	O Employee Assistance Program (EAP)
Item 2	O Programs to prevent or reduce stress
Item 3	O Alcohol or drug abuse support programs
Item 4	O Smoking cessation classes/counselling
Item 5	O Health Risk Assessment (HRA) – questionnaires about health habits
Item 6	O Self care books/tools
Item 7	O Physical activity and/or fitness programs
Item 8	O Nutrition education
Item 9	O Cholesterol reduction education
Item 10	O Weight management classes/counselling
Item 11	O Chronic disease management programs (e.g., diabetes, depression, cancer)
Item 12	O Screenings for high blood pressure
Item 13	O Screenings for cholesterol level
Item 14	O Screenings for any form of cancer
Item 15	O Screenings for diabetes
Item 16	O HIV or AIDS education
Item 17	O Nurse advice line
Item 18	O Balancing work and family education
Item 19	O Flexible hours for wellness
Item 20	O **None of the above** (mutually exclusive)

Does your company …. (please select all that apply)

Item 21	O Label healthy food choices in the cafeteria?
Item 22	O Offer special promotions/discounts to encourage healthy food choices?
Item 23	O Have onsite shower facilities?
Item 24	O Provide or encourage fitness breaks?
Item 25	O Have signage to encourage people to use the stairs?
Item 26	O Have fitness or walking trails on site?
Item 27	O **None of the above** (mutually exclusive)
